# Predictors of Undiagnosed Diabetes among Middle-Aged and Seniors in China: Application of Andersen’s Behavioral Model

**DOI:** 10.3390/ijerph18168396

**Published:** 2021-08-08

**Authors:** Chaozhou Mou, Minlan Xu, Juncheng Lyu

**Affiliations:** 1 Department of Mathematics Statistics, Shandong University, Weihai 264209, China; mouchaozhou@mail.sdu.edu.cn; 2 Department of Social Work, Shandong University, Weihai 264209, China; 3 Department of Public Health, Weifang Medical University, Weifang 261000, China; cheng_china@163.com

**Keywords:** diabetes, diagnosis, health service, Andersen’s behavioral model

## Abstract

Undiagnosed diabetes is a threat to public health. This study aims to identify potential variables related to undiagnosed diabetes using Andersen’s behavioral model. Baseline data including blood test data from the China Health and Retirement Longitudinal Study (CHARLS) were adopted. First, we constructed health service related variables based on Andersen model. Second, univariate analysis and multiple logistic regression were used to analyze the relations of variables to undiagnosed diabetes. The strength of relationships was presented by odds ratios (ORs) and 95% confidence intervals (CIs). Finally, the prediction of multiple logistic regression model was assessed using the Receiver Operating Characteristic (ROC) curve and the area under the ROC curve (AUC). According to diagnosis standards, 1234 respondents had diabetes, among which 560 were undiagnosed and 674 were previously diagnosed. Further analysis showed that the following variables were significantly associated with undiagnosed diabetes: age as the predisposing factor; medical insurance, residential places and geographical regions as enabling factors; having other chronic diseases and self-perceived health status as need factors. Moreover, the prediction of regression model was assessed well in the form of ROC and AUC. Andersen model provided a theoretical framework for detecting variables of health service utilization, which may not only explain the undiagnosed reasons but also provide clues for policy-makers to balance health services among diverse social groups in China.

## 1. Introduction

Diabetes increases the risks of cardiovascular complications and premature death in the general population, which not only causes tremendous economic and social burden, but also impacts the life quality of patients [1]. Diabetes has been increasing globally, both in developing and developed countries with population aging, urbanization and lifestyle changes [2,3]. However, nearly half of the worldwide diabetes cases were undiagnosed [4,5,6] and mainly distributed in developing countries [6,7]. Therefore, the diagnosed diabetes was described as just the tip of the iceberg [8].

In China, the prevalence of diabetes presented a noticeable rise as well [9,10,11]. The results from a national survey [4] indicated that diabetes had already reached epidemic proportions in the general adult population, in particular, the prevalence of undiagnosed diabetes and pre-diabetes were underestimated and would become a major threat to public health in the near future if no measure was taken [12]. Therefore, studies of undiagnosed diabetes are of importance to early diagnosis and the reduction in complications of diabetes.

Clinical studies have found various variables related to diabetes [13,14,15], which could be applied to prevent risk factors of diabetes as well as help screen undiagnosed diabetes [16], such as by using self-administrated risk score methods [17,18]. However, many previous studies mainly focused on exploring risk factors associated with disease instead of diagnosis availability [19], which were inadequate to understand why a great proportion of diabetes remains undiagnosed in the whole picture. Therefore, what factors impede accessible diagnosis are worth identifying.

Undiagnosed diabetes could be understood as a failure to access or seek health services [20]. There are some theories for studying the utilization of health services, among which Andersen’s behavioral model is most widely used as a theoretical framework to investigate the determinants of health service utilization [21]. The determinants are categorized into predisposing, enabling and need factors [22]. Predisposing factors include demographic characteristics, reflecting an individual’s propensity to use health services. Enabling factors are regarded as objective conditions that may facilitate or impede the use of health services. Need factors refer to the perceived and evaluated need for health services [23].

The progression of undiagnosed diabetes could lead to a serious and irreversible development of micro- and macro-vascular complications [24,25]. It was reported that the risk of mortality for undiagnosed diabetes was 1.5 to 3.0 times higher than that for the early diagnosed diabetes [26,27], indicating early diagnosis is crucial. In the wave 1 Irish Longitudinal Study of Ageing (TILDA), the Andersen framework was adopted to quantify the use of health services related to diabetes, despite the low prevalence of undiagnosed diabetes in the elderly Irish population [28,29]. However, few studies have explored undiagnosed diabetes from the view of health service utilization in China. Thus, in this study, we aim to (1) study undiagnosed diabetes among middle-aged and seniors under the theoretical framework of Andersen model, and (2) find out the potential factors related to the failed access to health service utilization. Results may provide clues for early diagnosis among undiagnosed diabetes.

## 2. Methods

### 2.1. Data Sources

The data used in this study was from the baseline data of China Health and Retirement Longitudinal Study (CHARLS), which was collected between May 2011 and March 2012 [30]. CHARLS provided a nationwide longitudinal survey on Chinese middle-aged and seniors, for the purpose of assessing their economic, social and health conditions [31]. Venous blood samples were also collected and assayed as part of the baseline survey [32]. The CHARLS baseline sample was recruited through the multistage probability-proportional-to-size (PPS) sampling technique [31]. Finally, respondents aged ≥ 45 and with blood tested were eligible for study, amounting to 11,587.

### 2.2. Definitions of Diagnosed and Undiagnosed Diabetes

Diagnosed diabetes was defined if the respondents answered ‘yes’ to the questions: “Have you been diagnosed with diabetes or high blood sugar?” in the questionnaire of CHARLS. Undiagnosed diabetes was defined if respondents answered ‘no’ to the above question but had fasting blood glucose ≥ 126 mg/dL, or random blood glucose ≥ 200 mg/dL, or glycosylated hemoglobin (HbA1c) ≥ 6.5% [33,34,35]. According to the definitions, the study population was divided into case group namely undiagnosed diabetes, and control group referring to previously diagnosed diabetes.

### 2.3. Health Service Related Variables

According to the Andersen model, the possible predictive variables were constructed from predisposing, enabling factors and need factors. The predisposing factors included gender, age (45–75/≥ 75), education level (literate/illiterate), being illiterate was defined as one could not read and write, and marriage status (having/no spouse or partner). The enabling factors comprised the following variables: household income level (average or above/below average), medical insurance (high/low reimbursement rate), which was dichotomized according to the fact that urban employee medical insurance and government medical insurance usually had a higher reimbursement rate than other listed insurances, medical facilities in the nearby (yes/no), residential places (urban/rural) and geographical regions (east/middle/west). Residential places and geographical regions were defined on the basis of division code by the National Bureau of Statistics [36]. Finally, the need factors included having other chronic diseases (no/yes) and self-perceived health status (good/fair/poor).

### 2.4. Data Analysis

A subset of CHARLS baseline data was set up in accordance with diagnosis outcome. First, frequencies of diagnosed and undiagnosed diabetes were presented by the health service related variables from the Andersen model. Second, the associations between health service related variables and diagnosis of diabetes were analyzed by using Pearson’s Chi-square test. Variables with a *p*-value < 0.2 were further analyzed in multiple logistic regression model [37]. Third, after checking for absence of multiple collinearity, the strength of associations was presented in terms of adjusted odds ratios (OR) and 95% confidence intervals (CI) based on multiple logistic regression analysis. Finally, the prediction capability of the regression model was assessed by the Receiver Operating Characteristic Curves (ROC) and the area under the ROC curve (AUC). The statistical significance was set at *p*-value < 0.05. Statistical software R 4.0 version was used in the data analysis [38].

## 3. Results

### 3.1. Sample Description

It was found that 5.82% (674/11,587) of the respondents reported having previously diagnosed diabetes, while 4.83% (560/11,587) of the respondents had diabetes undiagnosed. In total, 10.65% (1234/11,587) of the surveyed had diabetes, and 45.38% (560/1234) of all the diabetes were undiagnosed.

### 3.2. Diabetes Diagnosis by Health Service Related Variables in Univariate Analysis

A total of 1234 respondents had diabetes in this survey, among whom 44.17% (545/1234) were men. The average age was 59.46 years, and 8.51% (105/1234) patients with diabetes were older than 75. Regarding the education level, illiterate respondents accounted for 28.53% (352/1234) of the patients with diabetes. As to marriage status, 16.77% (207/1234) respondents with diabetes lived without a spouse or partner. Among the variables in predisposing factors, Table 1 presented that older adults had a higher percentage (55.24%) of undiagnosed diabetes than younger adults (44.46%). Based on the results of Chi-square test, it showed that age (*p* < 0.05) was statistically significant associated with the diagnosis of diabetes while gender, education level and marriage status (*p* > 0.05) were not (Table 1).

Among the variables in enabling factors (Table 1), respondents covered by medical insurance with the low reimbursement rate had a higher percentage (47.59%) of undiagnosed diabetes than those by insurance with the high reimbursement rate (33.84%). For the residential places, respondents living in the rural areas presented a higher percentage (51.38%) of being undiagnosed than those living in the urban areas (37.80%). So was the residential regions, respondents living in the west or middle had a higher percentage (54.92% and 43.22%, respectively) of being undiagnosed than those living in the east (38.74%). Further Chi-square test showed that medical insurance (*p* < 0.01), residential places (*p* < 0.0001) and geographical regions (*p* < 0.0001) had statistical significance in relation to diabetes diagnosis, whereas household income level and medical facilities (*p* > 0.05) did not (Table 1).

Among the variables in need factors (Table 1), patients with other chronic diseases had a lower percentage (39.35%) of undiagnosed diabetes than those with no other chronic diseases (66.30%). As for the self-perceived health status, poor and fair health status had a lower percentage (31.33% and 52.45%, respectively) of being undiagnosed than good health status (65.74%) did. Chi-square test further presented that having other chronic diseases (*p* < 0.0001) and self-perceived health status (*p* < 0.0001) both had statistical significance related to diabetes diagnosis (Table 1).

### 3.3. Variables Related to Diabetes Diagnosis in Multiple Logistic Regression Analysis

Having been checked for the absence of multiple collinearity among the covariates, variables with a *p*-value < 0.2 were further analyzed in the multiple logistic regression model [37], association strength of heath service variables with diabetes diagnosis was presented in adjusted odds ratio (OR) and 95% confidence intervals (CI). The results showed that old age (OR = 1.79, 95%CI: 1.16–2.77) compared to young age, medical insurance with the low reimbursement rate (OR = 1.64, 95%CI: 1.13–2.37) compared to that with the high reimbursement rate, living in rural areas (OR = 1.61, 95%CI: 1.24–2.11) compared to living in urban areas, living in the west (OR = 2.43, 95%CI: 1.78–3.30) or living in the middle (OR = 1.46, 95%CI: 1.08–1.96) compared to living in the east, were significantly related to undiagnosed diabetes with OR > 1, which increased risks to be undiagnosed (Table 2), while having other chronic diseases (OR = 0.41, 95%CI: 0.30–0.55) compared to no other chronic diseases and self-perceived health status as the poor (OR = 0.27, 95%CI: 0.19–0.38) or as fair (OR = 0.66, 95%CI: 0.47–0.94) compared to the self-perceived health status as good, were significantly related to undiagnosed diabetes with OR < 1, which increased protections from being undiagnosed (Table 2). However, gender, education level and medical facilities did not reach statistical significance in relation to diabetes diagnosis.

**Figure 1:**
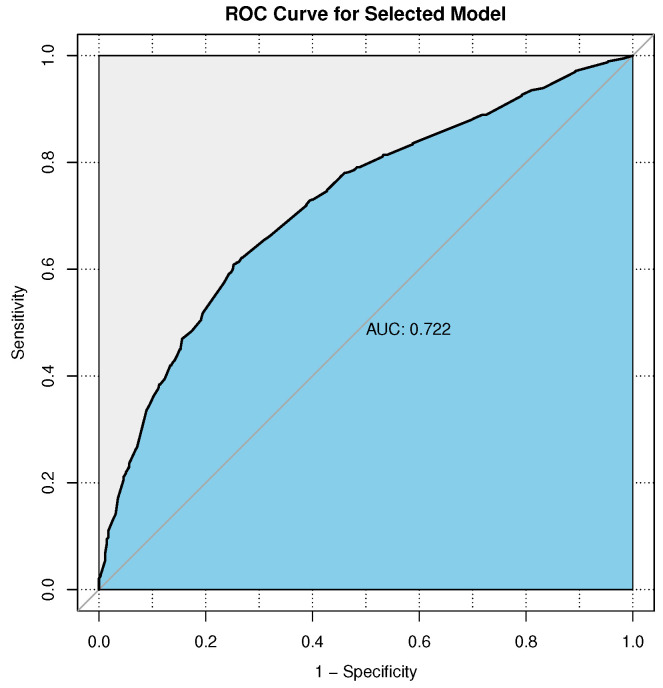
Receiver Operating Characteristics (ROC) curve and Area under the Curve (AUC) for the logistic regression model.

The ROC curve is a plot of sensitivity (no-diagnosis predicted to be no-diagnosis) versus specificity (diagnosis predicted to be diagnosis), which could be used to measure the model prediction. Figure 1 showed the ROC curve for the visualization of the predictive capability of the multiple logistic regression model. Considering that the area under the ROC curve (AUC) was 0.722, the goodness of fit was satisfactory.

## 4. Discussion

In this study, it was found that almost half (45.38%) of the respondents with diabetes remained undiagnosed, which was not a negligible fact. Based on the factors from the Andersen model, variables related to health service utilization were constructed and used to understand what types of social groups failed the diagnosis. The results disclosed that the elderly, those having medical insurance with low reimbursement rate, those living in rural areas, and those living in the economically underdeveloped middle or west China were at risk of diabetes undiagnosed. Whereas, people already being diagnosed with some other chronic diseases or self-perceived health status as being fair or poor were easy to have diabetes diagnosed.

Among the variables of predisposing factors from the Andersen model, age was the only significant variable associated with diabetes diagnosis, in particular, old seniors aged ≥ 75 increased risk odds of undiagnosed diabetes. Although previous studies have shown that prevalence of diabetes [4,12,13] including undiagnosed diabetes [13,39,40] increased with age, the effect of age on health service utilization was ambiguous [41], what may hinder the elderly in accessing health service utilization and diagnosis is worth further exploring. Some evidence showed that gender, education level and marriage status were related to health service utilization [41], but not in this study.

For the enabling factors, medical insurance with a low reimbursement rate was significantly associated with undiagnosed diabetes. Some studies have found that people covered by medical insurance were more likely to use health services [41,42], and may decrease the odds of undiagnosed diabetes [43]. In another study, more details were found that high deductibles and difficulty in reimbursement could result in low health service utilization among older people [44]. Our results further confirmed that owning medical insurance with the low reimbursement rate was at risk of having undiagnosed diabetes.

Previous studies found that the prevalence of diabetes among urban residents were higher than among rural residents in China [4,39,40]. In this study, it was found that living in rural areas was more likely to remain undiagnosed with diabetes. Some studies showed that the urban residents had higher likelihood of utilizing health services than rural residents [45,46], leading to more undiagnosed diabetes among rural residents. Several points may explain the urban-rural disparities. First, rural residents often had less education and health knowledge than urban residents [35]. Second, there was usually a shortage of health resources in rural areas [39]. Third, transportation to health institutions was limited in rural areas [35]. Moreover, living in poverty might be associated with few health care seeking behaviors [47]. In summary, the urban-rural difference in health knowledge level, health resources, convenience of transportation and socioeconomic status could be the barriers of health service utilization and result in the differences in diabetes diagnosis [45].

Similar urban-rural differential findings were also discovered in the geographical regions. In contrast to living in the east, living in the west or in the middle was more likely to have undiagnosed diabetes. Being rich in the east and poor in the west was thought to be the reason. Unbalanced economic development was closely correlated with unbalanced distribution of medical resources, including professional practitioners [48,49]. The results from one study presented that the distribution of doctors and nurses was highly uneven within or among provinces [50]. It is understandable that lack of professional human resources could impede health service utilization and further delay the diabetes diagnosis. In this study, whether there was medical facilities in the nearby was not related to diabetes diagnosis, implying that for human resources, health quality instead of quantity may play more important roles in early diagnosis.

Regarding the need factors, having other chronic diseases and self-perceived health status as fair or poor could significantly reduce the odds of undiagnosed diabetes, which were consistent with previous results [41,51]. People suffering chronic diseases had more chance of health service utilization [46]. Furthermore, contacting with the medical system could enhance the opportunities of diabetes diagnosis [40]. The more visits patients had, the earlier physicians may detect undiagnosed diabetes [40]. So was the situation with self-perceived health status, those who reported poorer health status were more likely to use health services and have diabetes diagnosed. Therefore, poor health status could possibly protect diabetes from being undiagnosed [41].

Each study has its weaknesses, there were also several limitations in this study. First, different definitions of diabetes may lead to different prevalence levels of diabetes. In this study, undiagnosed diabetes was defined by simultaneous use of HbA1c, fasting plasma glucose and random plasma glucose, which implies possible overestimation of cases and should be cautious in explanations and comparisons. Second, the results based on cross-sectional data could not be interpreted as being causal. Third, Andersen’s behavioral model has been continuously enriched and improved since it was proposed, which implies potential predictive variables in certain social or cultural backgrounds might not be included and could be further explored. In addition, the findings from the Chinese middle-aged and seniors could be different among other various populations, which is worthy of cross-nationally studying.

## 5. Conclusions

Failure in early diagnosis of diabetes could be explored in the perspective of health service utilization. For this, Andersen’s behavioral model provides a tool for detecting variables from the predisposing, enabling and need factors. This study presented that nearly half of patients with diabetes remained undiagnosed. Further analysis disclosed that patients who had the medical insurance with the low reimbursement rate, who resided in rural areas, and who lived in the west or middle China could predict undiagnosed diabetes, besides older age, health perception as poor or fair, and having other chronic diseases. In conclusion, undiagnosed diabetes can be predicted, which provides policy-makers not only with strategies about early diagnosis, but also confirms the necessity to balance health service utilization among the diversity of China’s social groups.

## Figures and Tables

**Table 1:** Univariate analysis of variables associated with diabetes diagnosis.

Variables	*n* (%)	Diabetes		Chi-Square *p* Value
		Undiagnosed (*N* = 560) *n* (%)	Diagnosed (*N* = 674) *n* (%)	
**Predisposing factors**				
**Gender**				0.13
Women	689 (55.83)	299 (43.40)	390 (56.60)	
Men	545 (44.17)	261 (47.89)	284 (52.11)	
**Age**				0.04
45–75	1129 (91.49)	502 (44.46)	627 (55.54)	
≥75	105 (8.51)	58 (55.24)	47 (44.76)	
**Education**				0.05
Literate	882 (71.47)	384 (43.54)	498 (56.46)	
Illiterate	352 (28.53)	176 (50.00)	176 (50.00)	
**Marriage status**				0.70
Having spouse or partner	1027 (83.23)	463 (45.08)	564 (54.92)	
No spouse or partner	207 (16.77)	97 (46.86)	110 (53.14)	
**Enabling factors**				
**Household income**				0.23
Average or Above	712 (57.70)	334 (46.91)	378 (53.09)	
Below average	522 (42.30)	226 (43.30)	296 (56.70)	
**Medical insurance**				0.0005
High reimbursement rate	198 (16.05)	67 (33.84)	131 (66.16)	
Low reimbursement rate	1036 (83.95)	493 (47.59)	543 (52.41)	
**Medical facilities**				0.12
Yes	894 (72.45)	393 (43.96)	501 (56.04)	
No	340 (27.55)	167 (49.12)	173 (50.88)	
**Residential places**				<0.0001
Urban	545 (44.17)	206 (37.80)	339 (62.20)	
Rural	689 (55.83)	354 (51.38)	335 (48.62)	
**Geographical regions**				<0.0001
East	413 (33.47)	160 (38.74)	253 (61.26)	
Middle	435 (35.25)	188 (43.22)	247 (56.78)	
West	386 (31.28)	212 (54.92)	174 (45.08)	
**Need factors**				
**Other chronic diseases**				<0.0001
No	276 (22.37)	183 (66.30)	93 (33.70)	
Yes	958 (77.63)	377 (39.35)	581 (60.65)	
**Perceived health**				<0.0001
Good	216 (17.50)	142 (65.74)	74 (34.26)	
Fair	469 (38.01)	246 (52.45)	223 (47.55)	
Poor	549 (44.49)	172 (31.33)	377 (68.67)	

**Table 2:** Variables related to undiagnosed diabetes in multiple logistic regression analysis.

Variables	Undiagnosed Diabetes		*p* Value
	OR	95%CI	
**Predisposing factors**			
**Age**			
45–75	Reference		
≥75	1.79 **	1.16–2.77	0.0087
**Enabling factors**			
**Medical insurance**			
High reimbursement rate	Reference		
Low reimbursement rate	1.64 **	1.13–2.37	0.0095
**Residential places**			
Urban	Reference		
Rural	1.61 **	1.24–2.11	0.0004
**Geographical regions**			
East	Reference		
Middle	1.46 *	1.08–1.96	0.01
West	2.43 **	1.78–3.30	<0.0001
**Need factors**			
**Other chronic diseases**			
No	Reference		
Yes	0.41 **	0.30–0.55	<0.0001
**Perceived health**			
Good	Reference		
Fair	0.66 *	0.47–0.94	0.02
Poor	0.27 **	0.19–0.38	<0.0001

* *p* < 0.05; ** *p* < 0.01.

## Data Availability

The data used in this study is publicly available, and can be accessed at Chinese Longitudinal Study on Health and Retirement (CHARLS) website http://charls.pku.edu.cn/index/zh-cn.html (accessed on 7 February 2021).
